# Estresse percebido em mulheres com síndrome metabólica: um estudo transversal*

**DOI:** 10.15649/cuidarte.2634

**Published:** 2023-05-28

**Authors:** Vinicius Santos-Barros, Wilkslam Alves-de-Araújo, Marcos Vinicius Santos-de-Jesus, Taynnan de Oliveira-Damaceno, Roseanne Montargil-Rocha, Josicélia Dumét-Fernandes, Randson Souza-Rosa, Isleide Santana Cardoso-Santo

**Affiliations:** 1 . Universidade Estadual do Sudoeste da Bahia (UESB), Jequié, BA, Brasil. Email: vinni.vieira@hotmail.com Universidade Estadual do Sudoeste da Bahia Universidade Estadual do Sudoeste da Bahia Jequié BA Brazil vinni.vieira@hotmail.com; 2 . Universidade Estadual do Sudoeste da Bahia (UESB), Jequié, BA, Brasil. Email: wilkslam@hotmail.com Universidade Estadual do Sudoeste da Bahia Universidade Estadual do Sudoeste da Bahia Jequié BA Brazil wilkslam@hotmail.com; 3 . Universidade Estadual do Sudoeste da Bahia (UESB), Jequié, BA, Brasil. Email: marcosvinicius.santos1710@ gmail.com Universidade Estadual do Sudoeste da Bahia Universidade Estadual do Sudoeste da Bahia Jequié BA Brazil marcosvinicius.santos1710@ gmail.com; 4 . Universidade Estadual do Sudoeste da Bahia (UESB), Jequié, BA, Brasil. Email: dtaynnan@gmail.com Universidade Estadual do Sudoeste da Bahia Universidade Estadual do Sudoeste da Bahia Jequié BA Brazil dtaynnan@gmail.com; 5 . Universidade Estadual do Sudoeste da Bahia (UESB), Jequié, BA, Brasil. Email: rmrocha@uesc.br Universidade Estadual do Sudoeste da Bahia Universidade Estadual do Sudoeste da Bahia Jequié BA Brazil rmrocha@uesc.br; 6 . Universidade Estadual do Sudoeste da Bahia (UESB), Jequié, BA, Brasil. Email: jodumet@hotmail.com Universidade Estadual do Sudoeste da Bahia Universidade Estadual do Sudoeste da Bahia Jequié BA Brazil jodumet@hotmail.com; 7 . Universidade Estadual de Feira de Santana (UEFS), Feira de Santana, BA, Brasil. Email: enfrandson@ gmail.com Universidade Estadual de Feira de Santana Universidade Estadual de Feira de Santana Feira de Santana BA Brazil enfrandson@ gmail.com; 8 . Universidade Estadual do Sudoeste da Bahia (UESB), Jequié, BA, Brasil. Email: isantana@uesb.edu.br Universidade Estadual do Sudoeste da Bahia Universidade Estadual do Sudoeste da Bahia Jequié BA Brazil isantana@uesb.edu.br

**Keywords:** Síndrome Metabólica, Dislipidemias, Obesidade Abdominal, Mulheres, Metabolic Syndrome, Dyslipidemias, Obesity, Abdominal, Women, Síndrome Metabólico, Dislipidemias, Obesidad Abdominal, Mujeres

## Abstract

**Introdujo:**

O estresse percebido foi sugerido como um fator de risco para o desenvolvimento da Síndrome Metabólica (SM). No entanto, pouco se sabe desta associaáo entre mulheres.

**Objetivo:**

Avaliar o estresse percebido em mulheres com SM.

**Materiais e Métodos:**

Estudo transversal, a partir do recorte de um ensaio clínico náo- randomizado, com pacientes de um centro de saúde público (RBR- 43K52N). A variável de desfecho foi a SM, utilizando os critérios do NCEP/ ATPIII. Foram coletados dados sociodemográficos, antropométricos, bioquímicos, hemodinamicos e aplicado da PSS.

**Resultados:**

A amostra constituiu-se de 75 mulheres acometidas por SM (47,69±8,15 anos de idade; 155,65±0,07 cm; 82,43±17,79 kg; IMC de 33,54±7,28). Encontrou-se valores elevados de RCQ entre as mulheres estressadas e náo-estressadas. A pontuaáo média da PSS foi de 27,73±9,17. Nos agrupamentos, verificou-se diferena significativa para PSS entre as mulheres estressadas e náo-estressadas (35,24±5,22 e 20,42±5,53, respectivamente; p=0,001). Observou-se também que mulheres estressadas tinham níveis mais elevados de triglicerídeos e níveis mais baixos de HDL-c quando comparadas com mulheres náo estressadas, embora sem diferena estatística.

**Discussoes:**

Os achados sugerem que as mulheres categorizadas como estressadas tinham maior pontuaáo da PSS, níveis mais elevados de triglicerídeos e níveis mais baixos de HDL-c quando comparadas com mulheres náo estressadas.

**Conclusoes:**

A pontuaáo da PSS foi significativamente maior entre mulheres com parametros lipídicos da SM alterados, com menores níveis de HDL- e aumento de triglicerídeos.

## Abstract (en)

**Introduction:**

Perceived stress has been suggested as a risk factor for the development of Metabolic Syndrome (MS). However, little is known about this association among women.

**Objective:**

To evaluate perceived stress in women with Metabolic Syndrome (MS).

**Materials and Methods:**

Cross-sectional study, based on a non-randomized clinical trial, with patients from a public health center (RBR-43K52N). The outcome variable was MS, using the NCEP/ATPIII criteria. Sociodemographic, anthropometric, biochemical, hemodynamic data and PSS application were collected.

**Results:**

The sample consisted of 75 women affected by MetS (47.69± 8.15 years old; 155.65±0.07 cm; 82.43±17.79 kg; BMI of 33.96±6 ,42). High WHR values were found among stressed and non-stressed women. The average PSS score was 27.73±9.17. In the groups, there was a significant difference for PSS between stressed and non-stressed women (35.24±5.22 and 20.42±5.53, respectively; p=0.001). It was also observed that stressed women had higher levels of triglycerides and lower levels of HDL-c when compared to non-stressed women, although without statistical difference.

**Discussions:**

The findings suggest that women categorized as stressed had higher PSS scores, higher levels of triglycerides and lower levels of HDL-c when compared to non-stressed women.

**Conclusions:**

the PSS score was significantly higher among women with altered MS lipid parameters, with lower levels of HDL- and increased triglycerides.

## Resumen

**Introducción:**

El estrés percibido ha sido sugerido como un factor de riesgo para el desarrollo del Síndrome Metabólico (SM). Sin embargo, poco se sabe sobre esta asociación entre las mujeres.

**Objetivo:**

evaluar el estrés percibido en mujeres con Síndrome Metabólico (SM).

**Materiales y Métodos:**

estudio transversal, basado en un ensayo clínico no aleatorizado, con pacientes de un centro de salud pública (RBR-43K52N). La variable resultada fue SM, utilizando los criterios NCEP/ ATPIII. Se recogieron datos sociodemográficos, antropométricos, bioquímicos, hemodinámicos y aplicación de PSS.

**Resultados:**

La muestra estuvo compuesta por 75 mujeres afectadas por SMet (47,69± 8,15 años; 155,65±0,07 cm; 82,43±17,79 kg; IMC de 33,96±6,42). Se encontraron valores altos de WHR entre mujeres estresadas y no estresadas. La puntuación media de PSS fue de 27,73±9,17. En los grupos, hubo una diferencia significativa para PSS entre mujeres estresadas y no estresadas (35,24±5,22 y 20,42±5,53, respectivamente; p=0,001). También se observó que las mujeres estresadas tenían niveles más altos de triglicéridos y niveles más bajos de HDL-c en comparación con las mujeres no estresadas, aunque sin diferencia estadística.

**Discusiones:**

Los hallazgos sugieren que las mujeres categorizadas como estresadas tenían puntajes PSS más altos, niveles más altos de triglicéridos y niveles más bajos de HDL-c en comparación con mujeres no estresadas.

**Conclusiones:**

la puntuación de PSS fue significativamente mayor entre las mujeres con parámetros lipídicos de SM alterados, con niveles de HDL más bajos y triglicéridos elevados.

## Introdujo

A síndrome metabólica (SM) é responsável por elevar o risco de doengas cardiovasculares e diabetes tipo 2. O diagnóstico da SM caracteriza-se pela presenta de pelo menos tres dos seguintes marcadores: obesidade abdominal, elevagáo da pressáo arterial, da glicemia de jejum, dos triglicerídeos e baixos níveis de lipoproteína-colesterol de alta densidade (HDL-c)^1^. Além desses marcadores, o estresse percebido foi sugerido como um fator de risco para o desenvolvimento da síndrome^2^. Geralmente, acredita-se que o sofrimento psicológico influencia diretamente na ocorrencia de resultados adversos a saúde, incluindo a SM e outras complicares de saúde.

Compreende-se o estresse como um processo de reagáo a uma demanda comportamental ou ambiental que limita a capacidade adaptativa de um individuo, resultando em alteragóes psicológicas e fisiológicas e ser um fator prognóstico para doengas e condigóes metabólicas^3^. O estresse percebido é altamente prevalente entre mulheres de meia-idade (entre 40-65 anos)^4^. As mulheres experimentam níveis de estresse percebido mais elevados do que homens, até mesmo quando se consideram os fatores demográficos e psicossociais^5^. Níveis mais altos de estresse em mulheres foram associados ao tabagismo, obesidade e diabetes tipo 2 e foi considerado um importante fator de risco para SM^6^^,^^2^.

As mulheres experimentam nessa fase da vida diversas situagóes de estresse, que incluem acúmulo de fungóes, relacionamentos, situagáo social, aspectos financeiros e desprazeres cotidianos que podem levar a níveis mais elevados de estresse percebido^7^. Portanto, estudos sugerem que a reagáo a situagóes estressoras é responsável pela elevagáo dos níveis de cortisol, como também pode estimular um padráo alimentar hipercalórico e comportamento sedentário, sendo que esses fatores contribuem potencialmente para o descontrole metabólico^8^. Padróes alimentares pouco saudáveis sáo os principais determinantes para o aumento da adiposidade e está associada a alteragóes negativas dos marcadores da SM^9^.

Assim, o estudo teve como objetivos avaliar o estresse percebido em mulheres com SM.

## Materiais e Método

### Desenho do estudo e populado

Realizou-se estudo transversal de um projeto de pesquisa maior aprovado pelo Comite de Ética em Pesquisa da Universidade Estadual do Sudoeste da Bahia (UESB, número CAAE 92352818.9.0000.0055, parecer: 2.850.239). Pacientes de um centro de saúde público na Atengáo Primária a Saúde, na zona urbana do município de Jequié (BA), foram recrutadas e avaliados entre os dia nove a 15 de abril de 2019 para avaliagáo clínica de um ensaio clínico náo-randomizado (REBEC, número RBR-43K52N). Para efeito do presente estudo as participantes eram adultas (>18 anos de idade) com diagnóstico de SM, conforme critérios do NCEP-ATP III^10^. Pacientes grávidas, com diabetes tipo I e comprometimento cognitivo foram excluídas do estudo. Setenta e cinco mulheres compuseram a amostra final para esta análise. Foi obtido consentimento por escrito de todas as participantes.

### Procedimentos

Inicialmente, a coleta de dados foi previamente agendada com os participantes por meio de ligagáo telefónica e realizada no centro de saúde por uma equipe de pesquisadores devidamente treinada do Grupo de Pesquisa Saúde e Qualidade de Vida da UESB. O diagnóstico da SM foi realizado de acordo com os critérios e recomendares definidas pelo NCEP-ATP III (Third Report of the National Cholesterol Education Program Expert Panelon Detection, Evaluation and Treatment of High Blood Cholesterol in Adults )^10^. A presenta da síndrome foi confirmada quando tres ou mais dos seguintes marcadores clínicos estavam presentes nas mulheres: medida da circunferencia abdominal >88 cm; pressáo arterial sistólica >130 mmHg e pressao arterial diastólica >85 mmHg; triglicerídeos >150mg/dl; <50 mg/dl e glicemia de jejum >100 mg/dl. As características das participantes, como idade (em anos completos), tabagismo, consumo de álcool, inatividade física, diagnóstico de hipertensao e diabetes tipo 2 foram coletadas por meio da aplicagáo de um questionário estruturado através de entrevistas individuais.

Os parámetros antropométricos de peso (kg) e estatura (cm) foram obtidos uma única vez por um mesmo pesquisador. O peso foi medido em uma balanga digital portátil (Wiso®, modelo W801), com precisáo de 0,1kg e capacidade máxima de 180kg. A estatura foi medida com um estadiometro metálico portátil (Sanny, modelo capriche), com precisao de 0,1mm e extensao máxima de 2m. Após mensuragao dessas medidas, calculou-se o índice de massa corporal (IMC= peso (kg)/altura x altura(m2). Conforme o valor do IMC, as mulheres foram classificadas como: eutróficas (18,5-24,9 Kg/m2); sobrepesadas (25,0 29,9 kg/m^2^; ou obesas (>30 kg/m^2^)^11^. Para obtengáo da relagáo cintura/quadril (RCQ), a circunferencia da cintura foi aferida na menor curvatura localizada entre as costelas e a crista ilíaca e a circunferencia do quadril na extensáo de maior protuberáncia glútea, com uma fita métrica flexível e inelástica com precisáo de 0,1 cm. O ponto de corte utilizado para RCQ foi de 0,80 cm^12^.

A medida da pressáo arterial sistólica (PAS) e diastólica (PAD) foi realizada com um aparelho semiautomático validado e calibrado Omron, modelo HEM-742 INT (Omron Healthcare, USA) e manguitos de tamanho adequados a circunferencia do brago das mulheres avaliadas (adulto pequeno (10x24), 22 a 26 cm; adulto (13x30), 27 a 34 cm; adulto grande (16x38), 35 a 44cm). As aferigóes foram feitas com as participantes na posigáo sentada, no brago esquerdo, após repouso de dez minutos. Todas elas foram instruídas a manterem-se relaxadas, apoiados contra o encosto da cadeira, com as pernas descruzadas e pés apoiados no cháo. Para a análise bioquímica, foi coletado 4 ml de sangue venoso na veia antecubital, após jejum confirmagáo de 12 horas, em sala de coleta preparada no centro de saúde. As amostras sanguíneas foram transferidas para tubos com gel separador, sem anticoagulante, devidamente identificados e armazenados em caixa térmica, em seguida, foram transportadas até o laboratório em um período máximo de duas horas. No laboratório, o soro foi separado por centrifugagáo durante dez minutos a 3.000 rpm sob uma temperatura de 6°C. As concentragóes séricas de glicose e níveis séricos de TG e HDL-c por métodos enzimáticos (Roche Diagnostics). A presenga de diabetes foi definida quando havia diagnóstico médico ou quando a glicemia em jejum era >126 mg/dl. A presenga de hipertensáo foi definida como PAS >140 mmHg e/ou PAD >90 mmHg ou utilizagáo medicagáo anti- hipertensiva^13^. A diabetes foi definida quando a glicemia em jejum era > 126 mg/dl, uso hipoglicemiante oral ou de insulina^14^.

A Escala de Estresse Percebido (PSS) foi utilizada para avaliar o estresse, desenvolvida por Cohen, Karmarck e Mermelstein (1983), traduzida e validada por Luft (2007)^15^. A PSS contém 14 itens, sendo sete positivos e sete negativos. Os itens negativos avaliam a falta de controle e as reagóes afetivas negativas, enquanto os itens positivos medem a capacidade de enfrentar as situagóes estressoras. Cada item é avaliado em uma escala de cinco pontos (0 = nunca; 1= quase nunca; 2= as vezes; 3= quase sempre; 4= sempre). A pontuagáo final varia de 0 a 56, mensurando situagóes de estresse nos últimos 30 dias. As pontuagóes variaram de 0 a 56, com pontuagóes mais altas indicando níveis mais altos de estresse percebido e as pontuagóes mais baixas indicando níveis mais baixos de estresse. Nesse estudo, a pontuagáo total da PSS foi dividida em duas categorias. A pontuagáo de corte utilizada foi de >28 pontos para categoria estressada e <28 pontos para categoria náo-estressada. Esse valor de corte foi selecionado de acordo com um estudo semelhante^16^.

### Análise estatística

Realizou-se frequéncia e estatística descritiva dos dados, foram reportados em média e desvio padrao ou mediana e quartis. A normalidade dos dados foi testada pelo Shapiro-Wilk test e o teste de Levene foi utilizado para verificar a homogeneidade das variancias. As características da amostra foram comparadas entre as mulheres estressadas e nao- estressadas usando o Teste t independente para as variáveis continuas e o Teste de Qui-quadrado para variáveis categóricas. Para avaliar a diferenga dos valores médios dos critérios da SM entre as mulheres estressadas e nao- estressadas, utilizou-se o teste t independente para medidas paramétricas e para as nao-paramétricas o teste de U de Mann-Whitney. O nível de significancia adotado foi p<0,05 e a análise estatística foi conduzida no software SPSS 22.0. O banco de dados foi armazenado no Mendeley Data^17^.

## Resultados

A amostra constituiu-se de 75 mulheres acometidas por SM, com idade média de 47,69 anos (DP= 8,15; 155,65±0,07 cm; 82,43±17,79 kg; IMC de 33,54±7,28). Encontrou-se valores elevados de RCQ na amostra geral e nos agrupamentos por nível de estresse percebido. A média geral de estresse percebido foi de 27,73±9,17. Nos agrupamentos, verificou-se diferenga significativa nas médias dos escores de estresse percebido entre as mulheres estressadas e nao-estressadas (35,24±5,22 e 20,42±5,53, respectivamente; p=0,001). Observa-se também que houve maior prevaléncia de hipertensao (82,7%), diabetes (70,7%) e inatividade física (54,7%). As características antropométricas e fatores de risco das mulheres sao apresentadas na Tabela 1.

**Tabela 1:** Características da amostra e das mulheres agrupadas como estressadas e nao estressadas com PSS Score 28 como o valor de corte. Jequié-BA, 2020

Características	Geral	Estressadas	Nao-estressadas
		n=37(49,3%)	n=38(50,7%)
Anos de idade	47,69±69	48,35±8,95	47,05±7,35
Altura (cm)	155,65±0,07	156,14±0,07	155,18±0,07
Peso (kg)	82,43±17,79	83,18±15,73	81,69±19,78
IMC (kg/m2)	33,54±7,28	33,29±7,70	33,78±6,95
Relagao cintura quadril	0,86±0,25	0,84±0,27	0,88±0,24
Escala de estresse percebido	27,73±9,17	35,24±5,22*	20,42±5,53
Tabagismo	17(22,7%)	6(16,2%)	11(28,9%)
Consumo de álcool	34(45,3%)	15(40,5%)	19(50,0%)
Inatividade física	41(54,7%)	18(48,6%)	23(60,5%)
Hipertensao	62(82,7%)	29(78,4%)	33(86,8%)
Diabetes tipo 2	53(70,7%)	25(67,6%)	28(73,7%)

Diferengas entre os grupos com o teste Qui-Quadrado para proposes e Teste t para amostras independentes. *p=0,001

A Tabela 2 mostra os valores de PSS e componentes das SM estudados nas mulheres agrupadas como estressadas e nao estressadas, com base na pontuagao de PSS <28 como o valor de corte. Em geral, as mulheres tiveram valores alterados em todos os componentes da SM em relagao aos critérios de diagnóstico da NCEP-ATP III. Observou-se que os valores de CA, PAS, PAD e Glicemia foram similares entre os agrupamentos. Entretanto, o nível sérico de triglicerídeos foi maior no agrupamento estressadas, como também apresentaram maior diminuido de HDL-c em relagáo as mulheres da categoría náo-estressadas. Contudo, esses valores náo foram estatisticamente significativos.

**Tabela 2:** Componentes da SM geral e das mulheres agrupadas como estressadas e nao estressadas com PSS Score <28 como o valor de corte. Jequié-BA, 2020

Variáveis	Geral	Estressadas	Nao-estressadas	
		n=37(49,3%)	n=38(50,7%)	^p^
CA (cm)	107,29±12,83	107,30±11,75	107,29±13,96	0,998
PAS (mmHg)	136,81±21,00	137,22±20,60	136,42±21,66	0,871
PAD (mmHg)	86,72±11,04	86,35±11,20	87,07±11,03	0,778
Glicemia (mg/dl)	143,26(89,00-180,00)	143,26(87,50-199,50)	143,26(99,25-171,75)	0,945
Triglicerídeo (mg/dl)	187,00(131,00-192,00)	189,67(137,50-241,50)	160,50(119,75-189,67)	0,354
HDL-c (mg/dl)	46,03(38,00-48,00)	44,00(40,00-46,03)	46,03(38,00-53,00)	0,077

*Diferengas entre os agrupamentos com teste t para amostras independentes e teste U-Mann Whitney para mediana (intervalo). CA: Circunferencia abdominal; PAS: Pressáo arterial sistólica; PAD: Pressáo arterial diastólica; HDL-c: HDL-colesterol (mg/dl).*

## Discussao

Nesse estudo, avaliamos o estresse percebido e os componentes da SM entre mulheres de um centro de saúde na Atengáo Primária a Saúde. As participantes, em sua maioria, eram obesas ou sobrepesadas, diabéticas, hipertensas, náo praticavam atividade física e com RCQ elevada (0,86±0,25). Foram categorizados em estressadas e náo-estressadas de acordo com pontuagáo obtida na escala de estresse percebido. Das 75 participantes, 37 (49,3%) mulheres estavam sob estresse. Os achados sugerem que as mulheres categorizadas como estressadas tinham maior pontuagáo da PSS, níveis mais elevados de triglicerídeos e níveis mais baixos de HDL-c quando comparadas com mulheres náo estressadas.

Sabe-se que a falta de atividade física aumenta as chances de acúmulo de gordura visceral em mulheres, e consequentemente, o risco de desenvolver doengas cardiovasculares^18^. Além disso, mulheres que vivenciaram situagóes estressantes estavam mais suscetíveis a desenvolverem hábitos náo saudáveis, como por exemplo: inatividade física e ingestáo alimentar excessivamente calórica^2^. Um estudo recente mostrou também uma relagáo significativa entre estresse e sintomas depressivos ou de compulsáo alimentar em mulheres^19^. A compulsáo alimentar é um transtorno mental comum incidente entre as mulheres, e parece aumentar a probabilidade de desenvolver marcadores específicos da SM, como a adiposidade abdominal^20^.

É interessante notar que cerca de 48,6% das mulheres estressadas náo praticavam atividade física, essa prevaléncia foi ainda maior entre mulheres náo-estressadas (60,5%). Em relagáo ao excesso de peso, a maioria das mulheres apresentaram sobrepeso (18,7%) ou obesidade (72%) diagnosticados pelo IMC e com circunferéncia abdominal elevada (107,29±12,83). O aumento da adiposidade abdominal foi considerado um preditor para dislipidemias e doenga cardiovascular^21^.

As mulheres tendem a expressar diferentes emogóes em situagóes de estresse. Por exemplo, viu-se que o enfrentamento das mulheres em relagáo ao aumento da pressáo arterial fez com que elas expressem com mais frequéncia sentimento de tristeza, ansiedade ou depressao^8^. Em nosso estudo, verificou-se que mulheres estressadas e nao-estressadas apresentaram alteragóes dos valores pressóricos (56,8% e 60,5%, respectivamente) e da glicemia de jejum (59,5% e 71,1%, respectivamente) e 82,7% das mulheres já tinham hipertensao e 70,7% apresentavam diabetes tipo 2. Um estudo com 1.243 adultos (67,2% mulheres), demonstrou que o estresse percebido foi associado a inatividade física, obesidade e desenvolvimento de aterosclerose^22^. Além disso, situagóes de estresse podem ser fator de risco potencial para hipertensao e doenga arterial coronariana^20^.

O estresse percebido tem sido associado a SM, por ocasionar alteragóes no controle do eixo hipotálamo-hipófise-adrenal, devido a inflamagao crónica ou uma interagao entre predisposigao genética e exposigao ao estresse^22^. Portanto, a incapacidade de um indivíduo de lidar com o estresse, ocasiona aumento da secregao de catecolaminas, aumentando os níveis de cortisol, o que por sua vez diminuem o controle do apetite e aumento da adiposidade abdominal, resultando na elevagao da pressao arterial, do acúmulo de lipídios e em resisténcia a insulina^23^. Assim, a exposigao ao estresse sem estratégias adequadas de enfrentamento pode configurar-se como um fator de risco potencial para SM.

É consolidado na literatura que a inflamagao induzida por estresse provoca acúmulo de gordura, promovendo alteragóes comportamentais e fisiológicas^8^. Mulheres estressadas apresentaram níveis significativamente mais elevados de neuropeptídeo Y (NPY) em comparagao com mulheres com baixo estresse^24^. O NPY tem potencial orexigénico e em situagóes de estresse estimula um padrao alimentar hipercalórico^25^. Demonstrou-se também que o estresse percebido mostra uma forte correlagao com o cortisol circulante, e ele, associado as catecolaminas, sao responsáveis pelo aumento do nível de glicose no sangue, e induzem resisténcia a insulina, um dos principais fatores na SM2.

Nosso estudo também mostrou que maior valor de triglicerídeos e menor nível de HDL-c eram mais comuns entre mulheres estressadas. Um estudo observou que tabagismo foi associado a baixos níveis de HDL-c, aumento dos triglicerídeos e maior circunferéncia abdominal^26^. Destaca-se que houve prevaléncia de 16,2% de fumantes entre as mulheres estressadas e 40,5% delas faziam consumo de álcool. Uma meta-análise verificou correlagóes entre estresse e níveis alterados dos triglicerídeos e HDL-c. Estudos indicam que a inatividade física também favorece a redugao do nível de HDL-c, o que parece sustentar nossos achados^2^^,^^27^.

A dislipidemia aterogénica (HDL-C baixa, hipertrigliceridemia) normalmente precede a manifestagao clínica completa dos marcadores específicos da SM e também foi associada a resisténcia a insulina^28^. Diante disso, o estresse percebido também mostra correlagao com dislipidemia^8^. Os baixos níveis plasmáticos de HDL-C e hipertrigliceridemia estao significativamente relacionados ao infarto do miocárdio em indivíduos com SM. Assim como, o acúmulo de lipídios pode desencadear um processo inflamatório nas paredes vasculares^29^.

Atualmente, a SM é considerada como uma doenga deletéria, multifatorial, assintomática, relacionada a inflamagao crónica capaz de colocar o indivíduo em vulnerabilidade por agregagao de marcadores de risco cardiovasculares^30^. É sabido que o estilo de vida adotado pela populagao brasileira atualmente, tem contribuído significativamente com a alta prevaléncia da SM, principalmente por ser uma síndrome invisível ao cotidiano das pessoas com doengas crónicas nao transmissíveis, onde muitos desconhecem seus fatores clínicos que facilitam seu diagnóstico^18^. Modificagóes no estilo de vida, como hábitos alimentares mais saudáveis (por exemplo, dieta mediterránea), praticar pelo menos 150 minutos semanais de atividade física de intensidade moderada, cessagao do tabagismo e do consumo alcoólico parecem diminuir o risco de desenvolver distúrbios relacionados a SM^19^.

Portanto, os achados do presente estudo realizado com 75 mulheres com SM indicam que 49,3% das participantes vivenciam situagáo de estresse, com pontuagáo da PSS significativamente maior quando comparada a outras mulheres com SM. Além disso, parece que o estresse percebido causa alteragáo dos parámetros lipídicos da SM. Considerando os resultados, as mulheres estressadas tiveram aumento dos triglicerídeos e baixo nível de HDL-c, em relagáo as mulheres da categoria náo- estressadas. Nossos resultados também sugerem que todos os fatores de risco mencionados acima devem ser considerados, de modo a (1) desenvolver programas de modificado no estilo de vida relacionados a saúde e (2) planejar mudanzas individuais e ambientais que considerem o estresse no contexto da SM, contribuindo assim para prevenir a propagado da síndrome e diminuir alteragóes dos seus marcadores.

A SM pode ser considerada como uma sindemia ou complexo sindromico, na qual uma ou mais alteragóes clínicas interagem entre si de forma sinérgica, sendo capaz de causar eventos cardiovasculares. Nessa perspectiva, sabe-se que a Atengáo Primária a Saúde se articula como um sistema de saúde que pode contribuir na prevengáo e controle da SM, uma vez que possui uma equipe interdisciplinar e multiprofissional em saúde. Sendo capaz de conduzir linhas de cuidados voltados para atengáo a saúde da pessoa com doenga cronica náo transmissível, e que pode garantir um cuidado clínico longitudinal as pessoas acometidas pela SM, visto que a SM se caracteriza como uma doenga cronica náo transmissível (DCNT) que necessita ser acompanhada periodicamente.

É importante reafirmar que a promogáo da saúde e o incentivo de mudangas de comportamentos de saúde deletérios como inatividade física, tabagismo, alcoolismo e o consumo de alimentos gordurosos sáo ferramentas terapéuticas náo farmacológicas essenciais para a redugáo da morbimortalidade ocasionada pela SM. A SM por ser considerada uma síndrome silenciosa, de difícil diagnóstico, negligenciada pelos profissionais da Atengáo Primária a Saúde, e que geralmente necessita da associagáo de outras medidas tais como a vigiláncia a saúde, bem como incremento de práticas cuidativas e educativas utilizadas na prevengáo e controle da SM.

Uma limitagáo deste estudo foi o pequeno tamanho amostral derivado de um delineamento transversal, a partir de um ensaio clínico náo-randomizado com participagáo voluntária restrita a adultos de um único centro de saúde. Portanto, os achados podem náo ser generalizáveis. As informagóes dietéticas das participantes náo foram investigadas. Contudo, nossos resultados parecem sugerir que pesquisas futuras investiguem as relagóes entre o estresse percebido e componentes específicos da SM, em especial os parámetros lipídicos.

## Conclusoes

A média da pontuagáo de estresse percebido foi significativamente maior entre mulheres com parámetros lipídicos da SM alterados. O estresse percebido parece estar associado com um nível mais alto de triglicerídeos e diminuigáo do HDL-c em mulheres com SM. Os resultados deste estudo enfatizam a necessidade de implementar programas para reduzir o estresse e melhorar o estilo de vida e outros fatores de risco de mulheres com SM.
